# Optimization of Extraction Conditions to Improve Chlorogenic Acid Content and Antioxidant Activity of Extracts from Forced Witloof Chicory Roots

**DOI:** 10.3390/foods11091217

**Published:** 2022-04-22

**Authors:** Morad Chadni, Emilie Isidore, Etienne Diemer, Otmane Ouguir, Fanny Brunois, Régis Catteau, Laurent Cassan, Irina Ioannou

**Affiliations:** 1 URD Agro-Biotechnologies Industrielles, AgroParisTech, CEBB, 51110 Pomacle, France; emilie.isidore@agroparistech.fr (E.I.); etienne.diemer@utc.fr (E.D.); otmane.ouguir@etu.univ-orleans.fr (O.O.); brunois.fanny@laposte.net (F.B.); irina.ioannou@agroparistech.fr (I.I.); 2 Laboratoire Transformations Intégrées de la Matière Renouvelable (UTC/ESCOM, EA 4297 TIMR), Centre de Recherche Royallieu, Université de Technologie de Compiègne, Sorbonne University Association, CS 60 319, CEDEX, 60203 Compiègne, France; 3 Association des Producteurs d’Endives de France (APEF), 2 Rue des Fleurs, 62000 Arras, France; regis.catteau@endive.fr (R.C.); laurent.cassan@endive.fr (L.C.)

**Keywords:** forced witloof chicory roots, *Cichorium intybus* L., chlorogenic acids, 3,5-*O*-di-caffeoylquinic acid, polyphenolic compounds, extraction, food by-products, ultrasound-assisted extraction, solid–liquid extraction, kinetic modelling

## Abstract

Chlorogenic acids are major phenolic constituents in many herbal medicines and exhibit various bioactivities that explain the growing interest in extracting chlorogenic acids from biomass. In this context, the present study aims to maximize 3-*O*-Caffeoylquinic acid (3-CQA) and 3,5-*O*-di-caffeoylquinic acid (3,5-diCQA) contents from forced witloof chicory roots and to analyze the extraction kinetic modelling. First, the solid–liquid ratio, ethanol concentration, extraction time and temperature were studied. The extraction conditions were optimized to maximize the extraction of these compounds. The maximum yields reached 5 ± 0.11 and 5.97 ± 0.30 mg/g dry matter (DM) for 3-*O*-Caffeoylquinic acid and 3,5-*O*-di-caffeoylquinic acid, respectively, in less than 6 min at 70 °C. Extraction with water as a solvent was assessed with the aim of proposing a second greener and less-expensive solvent. This extraction is very fast from 90 °C, with a maximum of 6.22 ± 0.18 mg/g_DM_ of 3-*O*-Caffeoylquinic acid, and instantaneous for 3,5-*O*-di-caffeoylquinic acid with a maximum of 6.44 ± 0.59 mg/g_DM_. In the second step, response surface methodology was employed to optimize the ultrasound-assisted extraction of antioxidants. The higher antioxidant activities were found at temperatures from 40 °C and at percentages of ethanol in the range of 35–70%.

## 1. Introduction

Chlorogenic acids belong to the phenolic acid class, one of the most-common categories of phenolic compounds in the plant kingdom. They have been the subject of increasing interest due to their beneficial bioactivities—mainly antioxidant and anti-inflammatory activities—which provide them with anti-cancer, anti-ageing or anti-osteoporosis properties [1,2,3,4]. The chlorogenic acids result from the esterification of one or several *trans*-cinnamic acids with quinic acid. They have axial hydroxyls on carbons 1 and 3 and equatorial hydroxyls on carbons 4 and 5. During the processing of the matrices, the *trans* isomers may be partially converted to *cis* isomers [5]. Coffee beans, chicory and sunflower revealed to be interesting sources of chlorogenic acids [6,7,8,9]. Cultivated chicory (*Cichorium intybus* L.) can be sorted into “industrial or root” groups for inulin extraction, the witloof group for chicon production (an etiolated bud eaten as salad) and the “pasture” group, derived from wild chicory. All morphological parts of chicory (roots, leaves, flowers) contain a large number of various chemicals [10]. Forced witloof chicory roots (FR), an agricultural by-product from chicon production, seems to be a promising source of chlorogenic acids. The predominating group of chlorogenic acids in chicory are represented by monocaffeoylquinic acids (mainly 3-*O*-caffeoylquinic acid) and 3,4-, 3,5- and 4,5-*O*-dicaffeoylquinic acids [8]. 3,5-*O*-dicaffeoylquinic acid has shown a very significant contribution to the antioxidant capacity of chicory root extracts [7].

Witloof chicory (*Cichorium intybus* L.) is a biennial plant cultivated in Europe. In France, approximately 160,000 tons of chicory are produced over 7400 hectares with 95% of the production occurring in the Hauts-de-France region. The production of chicon may be divided in two stages. The first stage aims to obtain well-tuberized roots by growing them in the fields from April–May to October–November. In the second stage, the chicon, an etiolated bud, is produced by forcing the roots in the dark for 21 days at 15–20 °C. Thus, the forced roots obtained after the chicons’ harvest are by-products, with only low value-added applications such as livestock feed supplements [11], biogas production [12] or composting before spreading in the field (personal communication of the Association des Producteurs d’Endives de France APEF). The extraction of chlorogenic acids from forced chicory roots would represent an interesting step in the whole process of the valorization of this by-product. In fact, the extraction of phenolic compounds before the use of forced witloof chicory roots (FR) as a feed supplement can be beneficial since phenolic compounds are considered antinutritional and must be eliminated. Similar valorizations of food by-products by the extraction of phenolic compounds have been reported in the literature for mustard seed meal [13,14], wood bark [15], grape pomace [16], brewer’s spent grains [17], wheat bran [18], apple pomace [19], olive cake [20] and food processing by-products [21].

The few studies on FR deal with the analytical characterization of the phenolic compounds contained in root extracts [8,22,23,24]; however, to our knowledge, there are no studies focusing on the optimization of the extraction process of chlorogenic acids from forced chicory roots. The extraction temperature, solvent composition and solvent-to-solid ratio are the main operating conditions which can influence the efficiency of the extraction process. Generally, extraction time is used as a factor, and only a few works have studied the evolution of phenolic compound yield as a function of time. However, it seems very important to follow the kinetics of the extraction of phenolic compounds in order to understand and to modulate the transfer mechanisms of the molecules; this information allows for the adjustment of the residence time and, thus, the reduction of energy consumption [25]. Water and organic solvents are generally used for the extraction of bioactive compounds from plant biomass [25]. Ethanol, which is considered a food-grade solvent, is the preferred organic solvent, especially when the extracts present a potential for applications in the pharmaceutical and food industries [26]. The use of water as an extraction solvent will make it possible to offer a green alternative solvent to ethanol; moreover, it is less expensive, facilitates the scaling up of processes and does not require an ATEX (explosive atmosphere) zone for its implementation.

Assessing the antioxidant activity of the extracts is important for process optimization. Indeed, several studies have shown that the antioxidant activity of extracts from plant biomass is not correlated to a specific phenolic compound, and that the optimal operating conditions may differ depending on the response to be optimized [27,28,29]. In the case of FR, several compounds, such as chlorogenic acids, sesquiterpene lactones [30,31] and other bioactive compounds, can contribute to the antioxidant activity of the extracts. Furthermore, the extraction of bioactive compounds can be improved using ultrasound due to acoustic cavitations produced in the solvent [32,33]. However, the feasibility of using ultrasound for the extraction of chlorogenic acids from FR has not been yet explored. Thus, the aim of this work is (i) to determine the influence of the main operating conditions of the extraction process (temperature of the extraction, solid–solvent ratio, solvent composition) on the yield of extracted chlorogenic acids; (ii) to study the extraction kinetics of chlorogenic acids under different extraction operating conditions; and (iii) to optimize the antioxidant activity of the FR extracts using response surface methodology.

## 2. Materials and Methods

### 2.1. Chemicals and Materials

Ethanol (99.9%), acetonitrile (99.9%) and formic acid (98–100%) were purchased from Thermo Fisher, (Illkirch, France). Ultra-pure water was obtained from a Milli-Q system (Millipore Corporation, Burlington, MA, USA). DPPH (2,2-Diphenyl-picrylhydrazyl) and Trolox^®^ (6-hydroxy-2,5,7,8-tetramethylchromane-2-carboxylic acid) were obtained from VWR, France. 3-*O*-caffeoylquinic acid standard (>98%) was purchased from TCI CHEMICALS (Zwijndrecht, Belgium); 4,5-di-*O*-caffeoylquinic acid (>90%) and 3,5-di-*O*-caffeoylquinic acid (>90%) were purchased from Sigma-Aldrich (Saint-Quentin-Fallavier, France).

### 2.2. Plant Material

**Figure 1:**
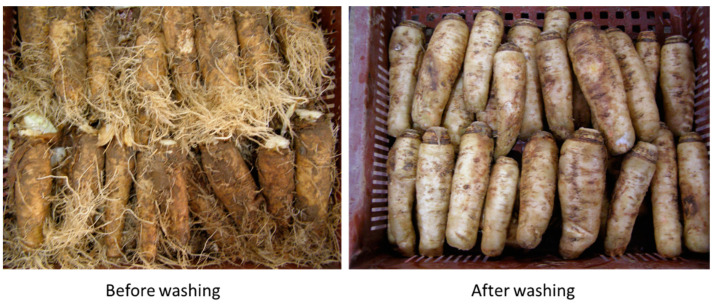
Forced witloof chicory roots before and after washing.

The APEF experimental station in Arras, France cultivated and kindly provided forced witloof chicory roots var. “Flexine–Vilmorin”. After the chicon harvest, the FR were washed with cold water (Figure 1).

Then, the roots were cut into slices and dried at 40 °C. The dried slices were ground using a commercial blender (WARING, VWR, Rosny-sous-Bois, France). The flour obtained was sieved and stored in a dark cold room at ambient temperature. The abundant fraction with a particle size lower than 500 µm was used for extraction.

### 2.3. Maximization of Chlorogenic Acid Content

#### 2.3.1. Study of the Effect of the Solvent Composition and the Solid–Liquid Ratio

Extractions were carried out using a shaking incubator (Thermo Scientific, Illkirch-Graffenstaden, France) for 24 h. An agitation speed of 180 rpm was applied at 30 °C. First, the effect of the solvent composition was studied using ethanol from 0 to 100% (*v*/*v*) in water, with a solid–liquid ratio of 1/50 (g_DM_/mL solvent). Second, the effect of the solid–liquid ratio was studied in the range of 1/10 to 1/200, using the best solvent composition found in the first step. The range for the solid–liquid ratio was selected based on preliminary experiments.

Samples were taken to be analyzed by UHPLC and to determine the chlorogenic acid content (mg/g_DM_).

#### 2.3.2. Kinetic Study of the Extraction of Chlorogenic Acids

The extraction of chlorogenic acids was carried out in an agitated glass reactor of 2 L. In all experiments, the agitation speed was fixed to 250 rpm and the solvent volume was 600 mL. The extraction temperature was regulated by a cryothermostat (Lauda, Germany).

The extraction kinetics were studied at different extraction temperatures (between 25 and 90 °C) for a period of 30 min, with 70% ethanol and water as solvents. Samples were taken at regular intervals. The liquid extract was separated from the solid residue by centrifugation (Allegra X15-R, Beckman Coulter, Villepinte, France) at 4713 g for 10 min at 4 °C. The supernatant was analyzed by UHPLC to determine the chlorogenic acid content (mg/g_DM_).

The extraction efficiency in different solvents and at different temperatures was compared by calculating the extraction rate and kinetic parameters of Peleg’s model [34]. The Peleg model was shown to be the most suitable for describing the experimental data of the extraction of phenolic compounds [35]. It is represented by Equation (1):

(1) $$Y\;\left(t\right)=y_{0}+\frac{t}{K_{1}+K_{2}\cdot t}$$

where Y (t) represents the concentration of chlorogenic acids at time t; $y_{0}$ is the concentration of chlorogenic acids at time *t* = 0; $K_{1}$ is Peleg’s rate constant (min g_DM_/mg) and$\;K_{2}$ is Peleg’s capacity constant (g_DM_/mg). Since $y_{0}$ in all experimental runs was zero, Equation (2) was used in the final form:

(2) $$Y\;\left(t\right)=\frac{t}{K_{1}+K_{2}\cdot t}$$

The Peleg rate constant $K_{1}$ relates to the extraction rate $R_{0}\;\left(\mathrm{mg}/g_{\mathrm{DM}}.\min\right)$ at the very beginning (*t* = $t_{0}$):

(3) $$R_{0}=\frac{1}{K_{1}}$$

The Peleg capacity constant$\;K_{2}$ relates to the maximum extraction yield, i.e., the equilibrium yield of extracted chlorogenic acids ($Y_{e}$). When *t* → ∞, Equation (4) gives the relations between the equilibrium yield and $K_{2}$ constant:

(4) $$Y\;{\left(t\right)}_{t\to \infty }=Y_{e}=\frac{1}{K_{2}}\;\;\;\;\;\left(\mathrm{mg}/g_{\mathrm{DM}}\right)$$

The kinetic parameters of Peleg’s model were calculated using Excel software (Version 2019) (Microsoft, Redmond, WA, USA). The accordance of the experimental yields and the model-predicted yields was evaluated by correlation coefficient (*R*^2^) and root mean square error (*RMSE*) (Equation (5)):

(5) $$RMSE=\sqrt{\frac{\sum_{i=1}^{n}{\left(y_{exp}-y_{pred}\right)}^{2}}{n}}$$

where n is the number of experimental points composing a kinetic curve; $y_{exp}$ is the experimental value at point i and $y_{pred}$ is the model value at point i.

#### 2.3.3. Determination of Chlorogenic Acid Content by UHPLC

A total of 1 mL of each extract was filtered through a 0.20 μm filter (Chromatofil filter, Xtra RC-20/25) before UHPLC analysis. 3-*O*-CQA, 4,5*-O*-di-CQA and 3,5-*O*-di-CQA were quantified by reversed-phase UHPLC-DAD (Ultimate 3000; Dionex, Sunnyvale, CA, USA, ThermoFisher, Waltham, MA, USA) equipped with a quadratic pump, an autosampler, a column furnace and a diode array detector. A gradient elution was performed using 0.1% formic acid (*v*/*v*) in water (solvent A) and acetonitrile (solvent B) on a Halo aQ Advanced Material Technology C18 column (Wilmington, BC, USA) (150 × 2.1 mm with 2.7 μm particle size). Solvent A’s gradient was as follows: 98% (0 min), 90% (3.5 min), 65% (6.5 min), 50% (7.5 min), 50% (8 min) and 98% (8.5 min), for a run of 10 minutes. The column temperature was set at 48 °C and the solvent had a constant flow rate of 0.8 mL/min. Chromatograms were acquired at 320 nm and processed with Chromeleon software (version 6.8). 3-*O*-CQA, 4,5-*O*-di-CQA and 3,5-*O*-di-CQA were identified by comparing their relative retention times with their respective standards.

### 2.4. Optimization of the Antioxidant Activity of Extracts by Response Surface Methodology

#### 2.4.1. Extraction Equipment and Procedure

The extraction was operated in an agitated glass reactor of 2 L, equipped with an ultrasound probe UP400St (400W, 24 kHz) and an ultrasound generator (Hielscher, Germany). The extraction temperature was monitored by a circulation of glycerol in an external jacket connected to a cryothermostat (Lauda, Germany). In all experiments, the agitation speed was fixed to 250 rpm, the time was 30 min and the solvent volume was 600 mL (S/L ratio = 1/100). The extractions were carried out using ultrasound in continuous mode at different amplitudes (20–100%).

#### 2.4.2. Experimental Design

The antioxidant activity was optimized using the response surface methodology (RSM). A Box–Behnken design was defined using the “MODDE” software (Version 12.0.1, Sartorius, Sweden). The design of the experiments consisted of 15 assays, including a triplicate at the central point (Table 1). The independent variables were the extraction temperature (25, 47.5 and 70 °C), the ultrasound amplitude (20, 60 and 100%) and the solvent composition (0, 50 and 100% ethanol in water). The reproducibility of the design was assessed by introducing central points in the design, which were repeated three times. The standard deviation (SD) was SD = 1.09. The response to be optimized is the antioxidant activity of the extracts, measured by DPPH assay.

The experiments were performed randomly to minimize the effects of unexplained variability. A second-order polynomial equation was used to fit the experimental data (Equation (6)):

(6) $$Y=\beta_{0}+\overset{k}{\underset{i=1}{\sum }}\beta_{i}X_{i\;}+\overset{k}{\underset{i=1}{\sum }}\beta_{\mathrm{ii}}X_{\mathrm{ii}\;}+\overset{k}{\underset{i=1}{\sum }}\overset{k}{\underset{j=i+1}{\sum }}\beta_{\mathrm{ij}}X_{\mathrm{ij}\;}+\varepsilon$$

where Y indicates the response; $\beta_{0}$ denotes the model intercept;$\;\beta_{i}$, $\beta_{\mathrm{ii}}$ and $\beta_{\mathrm{ij}}\;$represent the coefficients of the linear, quadratic and interactive effects, respectively; $X_{i}$ and $X_{j}$ are the coded independent variables; k is equal to the number of the tested factors (k = 3 in this study) and ɛ represents the differences between the experimental and the predicted values.

An analysis of variance (ANOVA) was then performed to test the model significance and adequacy towards the response. The model coefficients are considered as significant when the Student’s *t*-tests of significance results in a *p*-value below 0.05, which corresponds to a 95% confidence level.

### 2.5. Measurement of Antioxidant Activity by DPPH Assay

The radical scavenging capacity of the extracts was evaluated by measuring the absorbance loss at 515 nm, the maximal absorbance wavelength of DPPH• (2,2-Diphenyl-picrylhydrazyl). The colored DPPH• radical is reduced by the antiradical compounds contained in the tested solution. A standard solution of Trolox (6-hydroxy-2,5,7,8-tetramethyl-chroman-2-carboxylic acid) provides a referent antioxidant activity. Spectrophotometric analyses were performed according to [36]. For the determination of the radical scavenging activity, 1450 µL of DPPH• (0.06 mM in 96% methanol) was added to 50 μL of a suitable dilution of the sample extract or Trolox in methanol solution and agitated. The absorbance of the solution was measured at 515 nm [36] after 1h incubation in the dark at ambient temperature. The inhibition percentage of DPPH• was calculated following Equation (7):

(7) $$Inhibition\;\left(\%\right)=\frac{A_{Control}-A_{Sample}}{A_{Control}}\times 100$$

where $A_{Control}$ is the absorbance of the control at 515 nm and $A_{Sample}$ is the absorbance of the sample at 515 nm.

*Antioxidant activity*, which represents the response of the experimental design, was calculated using the calibration curve, giving absorbance versus Trolox concentrations (0.0125 mg/mL to 0.25 mg/mL) (Equation (8)). It was expressed as mg Trolox equivalent (TE) per g dry matter (mg TE/g_DM_):

(8) $$Antioxidant\;Activity\;\left(\mathrm{mg}\;\mathrm{TE}/g_{\mathrm{DM}}\right)=\frac{D\cdot C_{TE}\cdot V}{m_{DM}}$$

where D is the dilution, $C_{TE}$ is the concentration of the Trolox equivalent corresponding to the percentage of inhibition of the extract, V is the total volume of the extract and $m_{DM}$ is the mass of the sample based on dry mater.

### 2.6. Statistical Analysis

All experiments were conducted at least in duplicates. The standard deviations are represented by the error bars on each figure. The R software (4.1.0) [37] was used to perform an ANOVA (*p* < 0.05) in order to evaluate the significant effects of the ratio and ethanol percentages, and a Tukey test allowed us to find the significative differences between groups (represented by different letters on the figures).

## 3. Results and Discussion

The extraction process of chlorogenic acids was studied by determining the optimal operating conditions. Then, the residence time of the extraction was found by a kinetic study led at different temperatures in 70% ethanol and water. Finally, the antioxidant activity of extracts was optimized using surface response methodology and applying ultrasound in order to obtain high-antioxidant extracts.

### 3.1. Effect of the Solvent Composition

**Figure 2:**
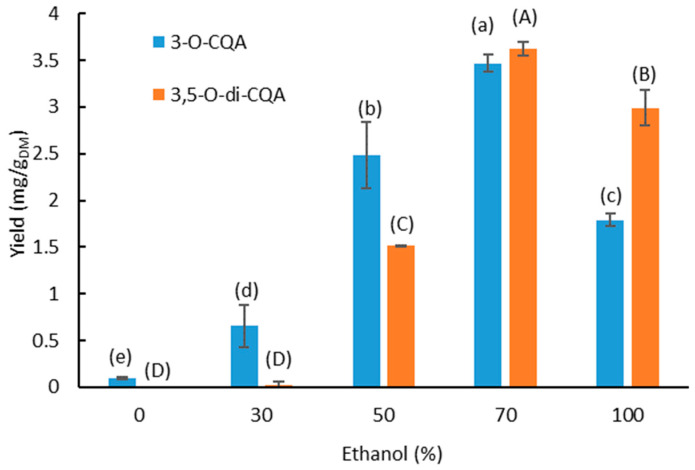
Effect of the percentage of ethanol in the extraction solvent on the extraction yield of 3-*O*-CQA and 3,5-*O*-di-CQA at (T = 30 °C; S/L = 1/50; t = 24 h). Bars with different small letters indicate significant differences in the yield of 3-*O*-CQA (*p* > 0.05) according to the Tukey test at 95%. Bars with different capital letters indicate significant differences in the yield of 3,5-*O*-di-CQA.

For food and/or pharmaceutical applications of chicory root extracts, water and ethanol were chosen as extraction solvents since they are considered non-toxic, food-grade, low cost and environmentally friendly [38]. Thus, water–ethanol mixtures with different percentages of ethanol were tested to find the optimal solvent composition to achieve the best extraction yields of 3-*O*-CQA and 3,5-*O*-di-CQA. Figure 2 shows the evolution of the extraction yield of the compounds according to different percentages of ethanol in water.

The extraction yields of both compounds follow a parabolic trend, with optimal values (3.47 ± 0.01 mg/g_DM_ and 3.62 ± 0.07 mg/g_DM_ for 3-*O*-CQA and 3,5-*O*-di-CQA, respectively) at 70% ethanol. For ethanol percentages above 70%, the extraction yields decrease by 48.5% and 17.67% for the mono-and di-caffeoylquinic acids, respectively. The extraction of the two molecules is more efficient with the ethanol–water mixture than with the pure solvents at 30 °C. Indeed, pure ethanol makes the extraction less efficient for both compounds (0 and 1.78 ± 0.01 mg/g_DM_ for 3,5-*O*-di-CQA and 3-*O*-CQA, respectively), and the use of pure water appears to be completely inefficient for both compounds (<0.09 ± 0.01 mg/g_DM_). The same results were found in the literature concerning the optimal percentage of ethanol to extract total phenolic compounds from chicory roots [8,39]. The mass transfer of phenolic compounds from the plant biomass into the liquid phase is controlled by their solubility in the extraction solvent. The solvent polarity has a major effect on this solubility. Thus, the selection of the solvents and their proportions, in the case of a mixture, is crucial for maximizing the transfer of the molecules from the matter towards the liquid phase. For further experiments, 70% ethanol was chosen.

### 3.2. Effect of the Solid–Liquid Ratio

**Figure 3:**
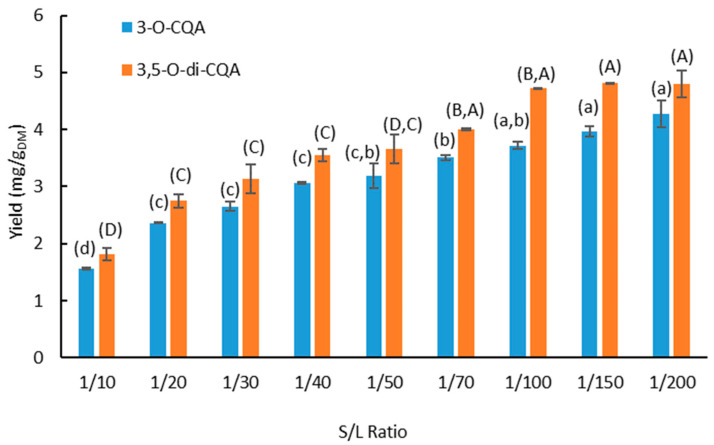
Effect of the solid–liquid ratio on the extraction yield of 3-*O*-CQA and 3,5-*O*-di-CQA (T = 30 °C; Solvent = 70% ethanol; t = 24 h). Bars with different small letters indicate significant differences in the yield of 3-*O*-CQA (*p* > 0.05) according to the Tukey test at 95%. Bars with different capital letters indicate significant differences in the yield of 3,5-*O*-di-CQA.

The effect of the solid–liquid ratio (S/L ratio) on the extraction yield of 3-*O*-CQA and 3,5-*O*-di-CQA was studied in the range of 1/10 to 1/200 (g dry matter/mL solvent). The results are presented in Figure 3.

The best extraction yields of the target compounds are achieved at a high S/L ratio and reach an equilibrium value from the ratio 1/100. Thus, the highest extraction yields obtained are 4.70 ± 0.01 and 3.70 ± 0.07 mg/g_DM_ for of 3-*O*-CQA and 3,5-*O*-di-CQA, respectively. When the ratios are set at 1/10, 1/20 and 1/30, the volume of the solvent is not sufficient to recover the chlorogenic acids from chicory roots. The solid–liquid ratio is one of the main operating parameters that requires study during the solid–liquid extraction. To achieve an efficient extraction, the solvent must permeate the plant matrix to reach the solutes. Therefore, the volume of the solvent must be sufficient to allow good hydration, adequate swelling of the plant matrix and a good diffusion of solutes to the extracellular medium [40,41]. The solvent volume must also be sufficient to avoid saturation of the solute in the liquid phase. On the other hand, an excessive volume of solvent will result in a very low concentration of the molecule of interest and high energy costs for the removal of this large volume of solvent. For further experiments, the solid–liquid ratio 1/100 was chosen.

### 3.3. Kinetic Study According to the Extraction Temperature

**Figure 4:**
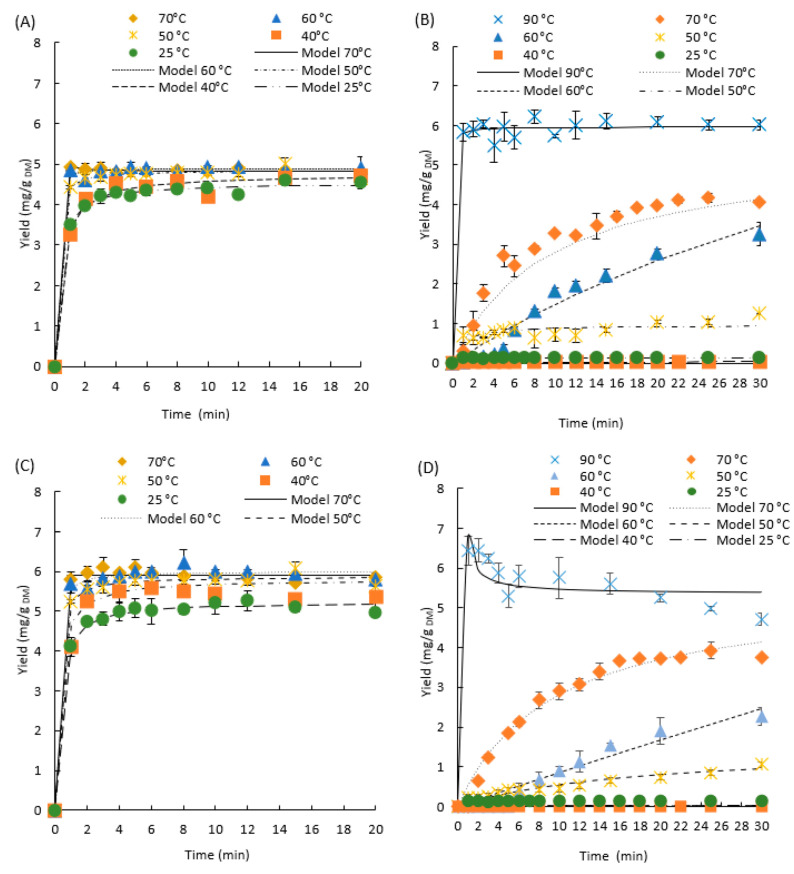
Effect of the temperature of extraction on the kinetics of the extraction of 3-*O*-CQA in 70% ethanol (**A**) and water (**B**) and 3,5-*O*-di-CQA in 70% ethanol (**C**) and water (**D**). (symbols—experimental yields; lines—approximation curves).

The extraction yields of 3-*O*-CQA and 3,5-*O*-di-CQA were followed according to the residence time, with two different extraction solvents (water, 70% ethanol) and at different temperatures. Water is used as a second, green and less-expensive extraction solvent. The extraction temperatures used with water vary between 25 °C and 90 °C, whereas, for 70% ethanol, the boiling point limits the tested temperatures to 70 °C. The kinetics are presented in Figure 4.

The effect of temperature on the extraction yield of 3-*O*-CQA and 3,5-*O*-di-CQA, as a function of time with 70% ethanol, is observed in Figure 4A,C, respectively. Under these conditions, the extraction of 3-*O*-CQA and 3,5-*O*-di-CQA is very rapid. The extraction equilibrium was reached in 1 min for temperatures superior or equal to 50 °C. The extracted chlorogenic acid contents, at the equilibrium, are equal to 5.0 ± 0.1 and 6.0 ± 0.3 mg/g_DM_ for 3-*O*-CQA and 3,5-O-di-CQA, respectively. For temperatures of 25 °C and 40 °C, the equilibrium was reached after 15 min of extraction. These conditions were not enough to reach the maximum yield of 3,5-*O*-di-CQA. The effect of temperature in the case of the extraction with 70% ethanol is largely dominated by the chemical effect of the hydroalcoholic solvent, which provides better solubilization and very rapid recovery of chlorogenic and 3,5-*O*-di-CQA, which is in accordance with the conclusions reported by Liu et al. in 2013. They demonstrated that the solvent composition played a significant role in the improvement of chicory roots’ extract yield, total polyphenolic compounds and antioxidant and antibacterial activities [39].

The results of the extraction with water as solvent are presented in Figure 4B,D. Unlike the extraction with 70% ethanol as the solvent, the extraction with water is strongly influenced by temperature. Indeed, at temperatures below 40 °C, the extraction of 3-*O*-CQA and 3,5-*O*-di-CQA is very low (<0.15 mg/g_DM_). From 50 °C, the extraction yield becomes proportional to the increase in temperature and extraction time. This extraction is very fast from 90 °C, with a maximum yield of 6.22 ± 0.18 mg/g_DM_ of 3-*O*-CQA reached in less than 8 min of extraction and 6.44 ± 0.59 mg/g_DM_ of 3,5-*O*-di-CQA reached within the first minute of extraction. The improvement in the aqueous extraction yields of 3-*O*-CQA at high temperatures confirms the effects of temperature on the modification of the physicochemical properties of water.

The experimental yields were compared with the predicted yields using Peleg’s model, as shown in Figure 4. The lines of the predicted yields passed through the most experimental yield points. The list of the estimated kinetic parameters, correlation coefficients and *RMSE*s is presented in Table 2.

The experimental data is highly fitted to the kinetic model if the values of the correlation coefficients are close to unity and if the *RMSE* is close to zero. In this work, the correlation coefficients ranged from 0.8466 to 0.9998 and the *RMSE*s from 0.0280 to 0.3580. In some conditions, the model was not valid due to instantaneous extraction (70% ethanol—70°C), to the degradation of 3,5-*O*-di-CQA (water—90 °C) and to the lower yields of the extraction (water—40 °C, 25 °C).

The values of the extraction rate constant (K_1_) and the constant of the extraction extent (K_2_) exhibited a tendency of decrease with increasing extraction temperature, indicating a higher initial extraction rate. With 70% ethanol as the solvent, the highest values for the initial extraction rates (R_0_) were obtained at 60 °C (204.08 ± 12.54 and 77.51 ± 18.52 mg/g_DM_ min for 3-*O*-CQA and 3,5-*O*-di-CQA, respectively). When water was used as the solvent, the highest initial extraction rates were obtained at 90 °C for 3-*O*-CQA (R_0_ = 197.07 ± 0.01 mg/g_DM_ min) and at 70 °C for 3,5-*O*-di-CQA (R_0_ = 0.58 ± 0.05 mg/g_DM_ min).

In same manner, the positive effect of temperature on the maximal yield of extraction (1/K_2_) was observed. The influence of extraction temperature was stronger with water as the solvent than with 70% ethanol, where at T = 70 °C, the equilibrium yield of extraction $\left(Y_{e}\right)$ was 5.34 ± 0.32 and 5.45 ± 0.01 mg/g_DM_ for 3-*O*-CQA and 3,5-*O*-di-CQA, respectively, while at T = 50 °C, the equilibrium yield of extraction $\left(Y_{e}\right)$ was 0.96 ± 0.02 and 1.48 ± 0.14 mg/g_DM_ for 3-*O*-CQA and 3,5-*O-*di-CQA, respectively.

**Figure 5:**
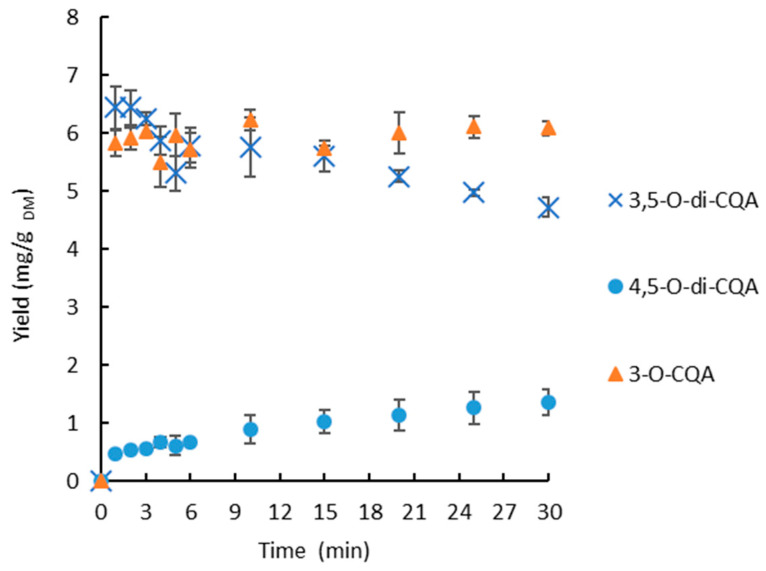
Thermal stability of CGAs in aqueous chicory roots extract at 90 °C.

At a temperature of 90 °C, an important decrease of the yield of 3,5-*O*-di-CQA was noticed during the extraction (Figure 4D), whereas 3-*O*-CQA was stable in these conditions. The thermal instability rate was about 7.7 × 10^−3^ mg/g_DM_ per min. The decrease 3,5-*O*-di-CQA is certainly due to a degradation of this molecule. By observation of the HPLC chromatograms, the apparition of its isomer, the 4,5-*O*-di-CQA, was observed. Thus, it seems interesting to study the stability of these 3 CGAs at the temperature of 90 °C (Figure 5).

**Figure 6:**
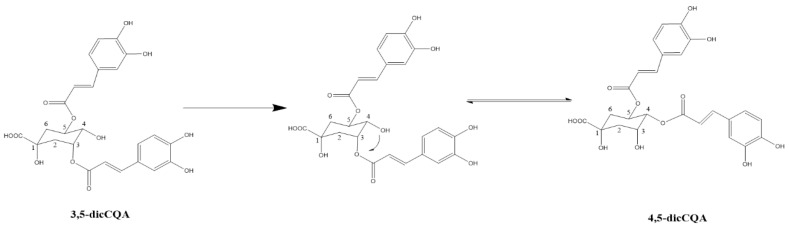
Isomerization reaction of 3,5-*O*-di-CQA to 4,5-*O-*di-CQA.

The amount of 4,5-*O*-di-CQA produced is proportional to the rate of decrease for 3,5-*O*-di-CQA, which confirms the transformation of this latter to 4,5-*O*-di-CQA by an acyl migration, as shown in Figure 6.

The results observed in Figure 5 confirm that temperature has a great effect on the stability of dicaffeoylquinic acids. This has been reported by many authors [42,43]. In fact, Li et al., in 2015, determined a ranking of dicaffeoylquinic acids based on their thermal stability upon heating in a boiling water bath. Thus, 4,5-*O*-di-CQA is more stable than 3,5-*O*-di-CQA, with the most sensitive being 3,4-*O*-di-CQA. Di-CQAs can isomerize or degrade to mono-CQAs, caffeic acid and other compounds [44]. The isomerization might be due to the fact that di-acyl CQAs are more stable when the ester bond with the quinic acid exists as an equatorial, rather than as an axial bond [43,45]. Only one ester bond is in the equatorial position for 3,4-*O*-di-CQA and 3,5-*O*-di-CQA, while for 4,5-*O*-di-CQA, both ester bonds are.

### 3.4. Optimization of the Antioxidant Activity of Extracts

A Box–Behnken experimental design was employed to maximize the antioxidant activity of the extracts. The design comprises 15 trials from 3 independent variables at 3 levels of the system: temperature (25, 47.5 and 70 °C), ultrasound amplitude (20, 60 and 100%) and solvent (0, 50 and 100% ethanol in water). The ANOVA was applied to analyze the experimental data and study the statistical significance and regression coefficients of the model terms (Table 3).

Coefficients are considered significant for *p* < 0.05. Thus, the temperature and quadratic term for ethanol percentages were found to be significant factors (*p* < 0.05) for predicting the antioxidant activity of the extracts, whereas the ultrasound amplitude was found to be non-significant (*p* > 0.05). Non-significant coefficients were removed to obtain a reduced model. The adequacy of the model was evaluated using the values of the coefficients of determination: *R*^2^ and adjusted *R*^2^ values. The analysis of variance shown in Table 3 indicates a good model fit, with a *R*^2^ value of 0.973 and an adjusted *R*^2^ value of 0.965. The model’s validity and reproducibly are high. The total analysis of the model is given in the Supplementary Materials (Figures S1–S4).

A second-order polynomial equation was used to build a mathematical model to find the optimal operating conditions for maximizing antioxidant activity. The second-order model developed in terms of coded variables is given in (Equation (9)):

(9) $$Antioxidant\;activity\;\left(\mathrm{mg}\;\mathrm{TE}/g_{\mathrm{DM}}\right)=20.354+0.445\cdot Eth+1.475\cdot Temp-8.502\cdot Eth\cdot Eth$$

**Figure 7:**
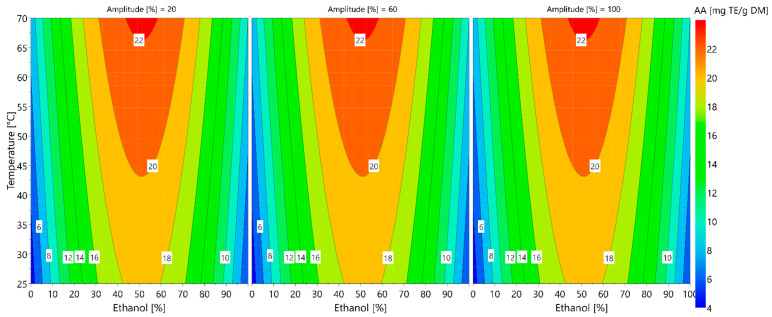
The 4D contour plots of the response, describing the antioxidant activity (AA) of the extract according to the extraction temperature, ethanol percentage and ultrasound amplitude.

The 4D contour plot of the response is shown in Figure 7.

The 4D contour plots show that the highest antioxidant activities (>20 mg Trolox/g_DM_) are obtained for temperatures superior to 40 °C and for percentages of ethanol between 35 and 70%. These operation conditions also allow the highest extraction yields of chlorogenic acids to be obtained. These results are in agreement with those of Lavelli, who, in 2008, studied red chicory products (*Cichorium intybus* L.var. silvestre). The author found that the total amount of extracted phenolic compounds is correlated with the antioxidant activity of the extracts measured by assays, based on free radical scavenging and enzyme inhibition [46].

Ultrasounds have no significant effect on the antioxidant activity of the extracts. Indeed, the chicory root cells could have already been damaged during the pre-treatment of the matter (the drying and grinding processes); thus, the ultrasounds have no complementary effect on the already-degraded cells. The same findings were drawn by Zardo et al. in 2019, during the ultrasound-assisted extraction of chlorogenic acids from sunflower seed cake [47]. The authors found that ultrasounds had a smaller influence on extraction yield than ethanol concentration and temperature, probably because plant cells had already been damaged by the oil extraction process.

Table 4 shows the antioxidant activity of extracts obtained under the optimum conditions of extraction. The predicted conditions for obtaining the maximum antioxidant activity were at 70 °C with 50% ethanol as the solvent. Experimental analyses of the antioxidant activity of the extracts obtained using the optimized extraction method were compared with the predicted response. The experimental and the predicted results were close, which validated the model.

## 4. Conclusions

Forced witloof chicory roots, by-products of chicon agricultural production, can be valorized for their 3-*O*-CQA and 3,5-*O*-di-CQA content. The experiments carried out in this paper have shown that the optimal extraction operating conditions were obtained either with 70% ethanol at a temperature of 50 °C or 100% water at a temperature of 90 °C. The extraction kinetics showed that the process duration may be as short as 1 min in water at high temperature. However, the thermal stability of the 3,5-*O*-di-CQA is a point to take into account for the optimization steps. A life-cycle assessment of these two processes (hydro–ethanolic and aqueous extraction) to determine which one is the best from an ecological perspective would be interesting. Extracts obtained from forced chicory roots have an antioxidant activity, which confers them interesting applications in the food industry, such as to produce healthy food, or in the pharmaceutical industry. The addition of ultrasound does not seem relevant, as long as the material has undergone pre-treatment such as drying and grinding. Additional studies considering the environmental impact and genetic variability of witloof cultivar and the cost of production will have to be carried out in order to determine the optimal conditions for the production of CGAs-based extracts.

## Figures and Tables

**Table 1:** Variables and levels used in the Box–Behnken design.

	Factors Level (Code)			Response
Run Order	Amplitude	Ethanol	Temperature	mg Trolox/g_DM_
**7**	20 (−1)	0 (−1)	47.5 (0)	4.52
6	100 (+1)	0 (−1)	47.5 (0)	4.18
14	20 (−1)	100 (+1)	47.5 (0)	6.54
9	100 (+1)	100 (+1)	47.5 (0)	7.35
8	20 (−1)	50 (0)	25 (−1)	17.53
2	100 (+1)	50 (0)	25 (−1)	17.24
1	20 (−1)	50 (0)	70 (+1)	24.78
4	100 (+1)	50 (0)	70 (+1)	21.98
15	60 (0)	0 (−1)	25 (−1)	5.62
3	60 (0)	100 (+1)	25 (−1)	3.25
10	60 (0)	0 (−1)	70 (+1)	5.30
11	60 (0)	100 (+1)	70 (+1)	7.19
13	60 (0)	50 (0)	47.5 (0)	20.79
12	60 (0)	50 (0)	47.5 (0)	19.11
5	60 (0)	50 (0)	47.5 (0)	21.18

**Table 2:** Values of Peleg’s constants with correlation coefficient $R^{2}\;$and the root mean square error (*RMSE*) in 70% ethanol and water at different temperatures.

Chlorogenic Acid	Solvent	Temperature °C	K_1_ (min g_DM_/mg)	K_2_ (g_DM_/mg)	*R* ^2^	*RMSE*
3-*O*-CQA	70% Ethanol	70	NV	NV	NV	NV
		60	0.0049 ± 0.0003	0.2043 ± 0.0032	0.9984	0.0763
		50	0.0194 ± 0.0007	0.2061 ± 0.0044	0.9982	0.0786
		40	0.0834 ± 0.0051	0.2105 ± 0.0078	0.9933	0.1642
		25	0.0622 ± 0.0020	0.2194 ± 0.0008	0.9979	0.0787
	Water	90	0.0051 ± 0.0009	0.1677 ± 0.0013	0.9936	0.1724
		70	1.3101 ± 0.1833	0.1875 ± 0.0366	0.9869	0.3218
		60	5.7985 ± 0.0001	0.0947 ± 0.0002	0.9782	0.2538
		50	0.9648 ± 0.4278	1.0397 ± 0.3028	0.8466	0.1471
		40	NV	NV	NV	NV
		25	NV	NV	NV	NV
3,5-*O*-di-CQA	70% Ethanol	70	NV	NV	NV	NV
		60	0.0129 ± 0.0166	0.1662 ± 0.0043	0.9973	0.1214
		50	0.0202 ± 0.0002	0.1701 ± 0.0045	0.9983	0.0934
		40	0.0405 ± 0.0021	0.1722 ± 0.0984	0.9908	0.2383
		25	0.0473 ± 0.0045	0.1908± 0.0845	0.9977	0.0944
	Water	90	NV	NV	NV	NV
		70	1.7362 ± 0.2126	0.1832 ± 0.0008	0.9903	0.2038
		60	11.6290 ± 0.0480	NV	0.9703	0.2230
		50	11.8233 ± 0.4521	0.6735 ± 0.0766	0.9694	0.0680
		40	NV	NV	NV	NV
		25	NV	NV	NV	NV

NV means non-validated data due to the very low yield of extraction, the isomerization of chlorogenic acids or to the instantaneous extraction (not enough points to draw the curve).

**Table 3:** Model equation coefficients and statistical parameters.

	Coefficient	*p*-Value
Constant	20.354	<0.001
Amplitude (Amp)	−0.250	0.632 NS
Ethanol (Eth)	0.445	0.286 NS
Temperature (Temp)	1.475	0.003
Amp × Amp	0.095	0.868 NS
Eth × Eth	−8.502	<0.001
Temp × Temp	−0.081	0.888 NS
Amp × Eth	0.166	0.764 NS
Amp × Temp	−0.362	0.520 NS
Eth × Temp	0.614	0.292 NS
ANOVA:		
*R*^2^ = 0.973		
*R*^2^ adj. = 0.965		

NS = Non-significant.

**Table 4:** Optimal extraction conditions and validation of the experimental and predicted antioxidant activity obtained under the same conditions.

Response Variable	Optimum Extraction Conditions			Maximum Values (mg TE/g_DM_)	
	Ethanol (%)	Temperature (°C)	Amplitude (%)	Predicted	Experimental
Antioxidant activity of extracts (mg TE/g_DM_)	50	70	0	21.80 ± 1.09	25.88 ± 0.29

## Data Availability

The data presented in this study are available in this article and supplementary materials.

## Supplementary Materials

The following supporting information can be downloaded at: https://www.mdpi.com/article/10.3390/foods11091217/s1: Figure S1. Summary of fit plot; Figure S2. Coefficient plot after removing the insignificant terms; Figure S3. The correlation between the observed and the predicted responses; Figure S4. Residual normal probability.
