# Supplementary material for: Optimization of Extraction Conditions to Improve Chlorogenic Acid Content and Antioxidant Activity of Extracts from Forced Witloof Chicory Roots
Source: Foods. 2022 Apr 22;11(9):1217. doi: 10.3390/foods11091217 (PMC9102191; doi:10.3390/foods11091217)
Supplement: Supplementary file 1 [file foods-11-01217-s001.zip › foods-1680647-supplementary.pdf]

Supplementary data

Optimization of Extraction Conditions to Improve Chlorogenic Acid Content and Antioxidant Activity of Extracts from Forced Witloof Chicory Roots

Morad Chadni [1,*], Emilie Isidore [1], Etienne Diemer [1,2], Otmane Ouguir [1], Fanny Brunois [1], Régis Catteau [3], Laurent Cassan [3] and Irina Ioannou [1,*]

[1] URD Agro-Biotechnologies Industrielles, CEBB, AgroParisTech, 51110, Pomacle, France ; morad.chadni@agroparistech.fr (M.C); emilie.isidore@agroparistech.fr (E.I); otmane.ouguir@etu.univ-orleans.fr (O.O) ; fanny.brunois@agroparistech.fr (F.B); irina.ioannou@agroparistech.fr (I.I)

[2] Sorbonne universités, Université de technologie de Compiègne, Laboratoire Transformations Intégrées de la Matière renouvelable (UTC/ESCOM, EA 4297 TIMR), Centre de recherche Royallieu, CS 60 319, 60 203 Compiègne Cedex, France ; etienne.diemer@utc.fr (E.D)

[3] Association des Producteurs d'Endives de France, APEF, 2 Rue des Fleurs, 62000 Arras, France ; laurent.cassan@endive.fr (L.C) ; regis.catteau@endive.fr (R.C)

* Correspondence: morad.chadni@agroparistech.fr (M.C) ; Tel +33 (0) 3 52 62 04 67
irina.ioannou@agroparistech.fr (I.I) ; Tel +33 (0) 3 52 62 04 68

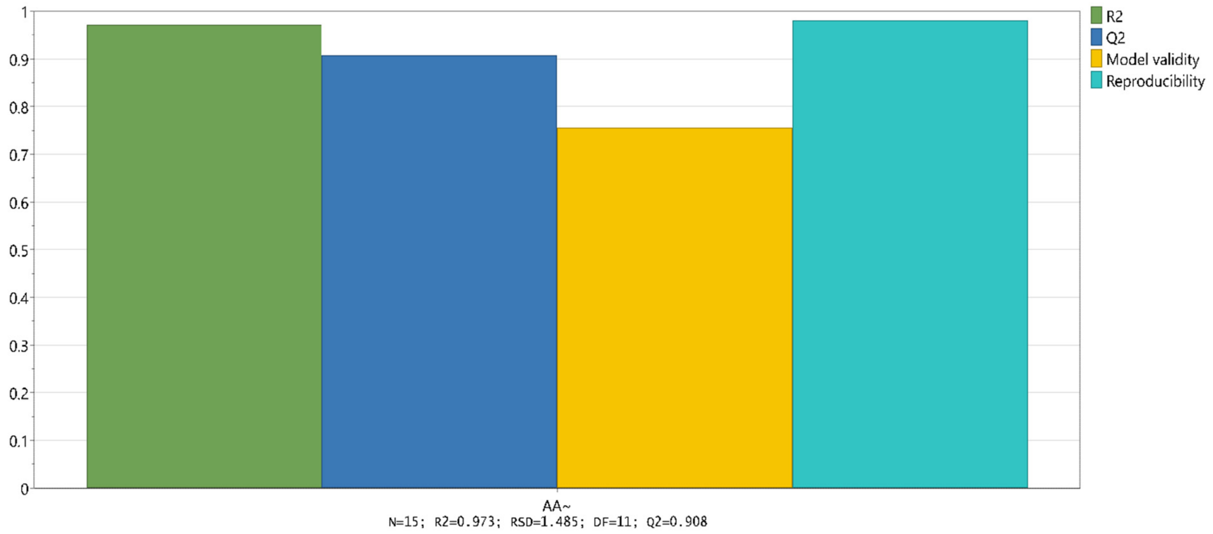

Figure S1. Summary of Fit Plot

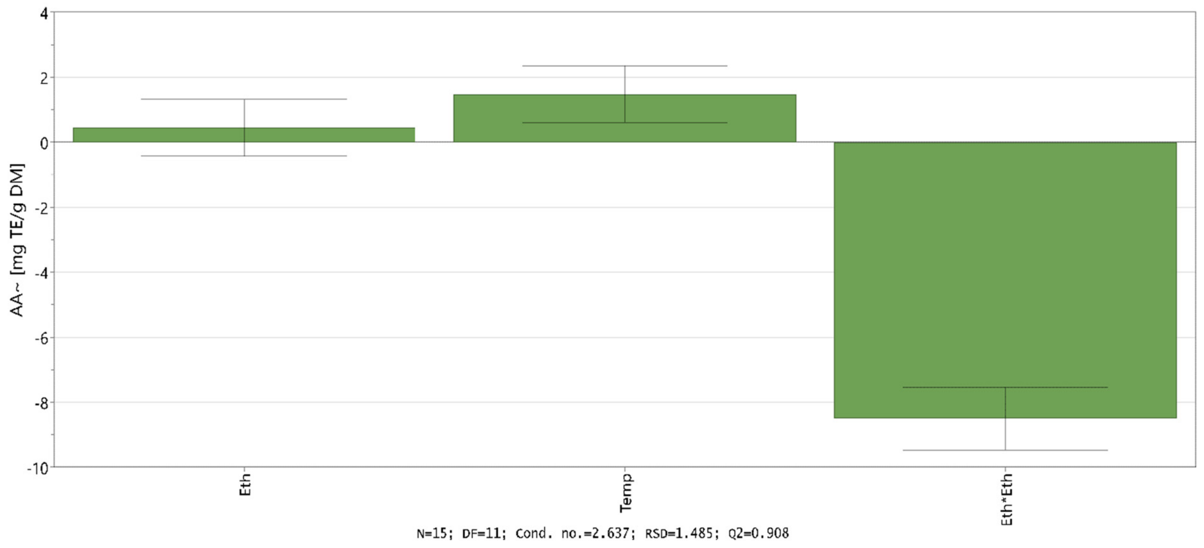

Figure S2. Coefficient Plot after removing the insignificant terms

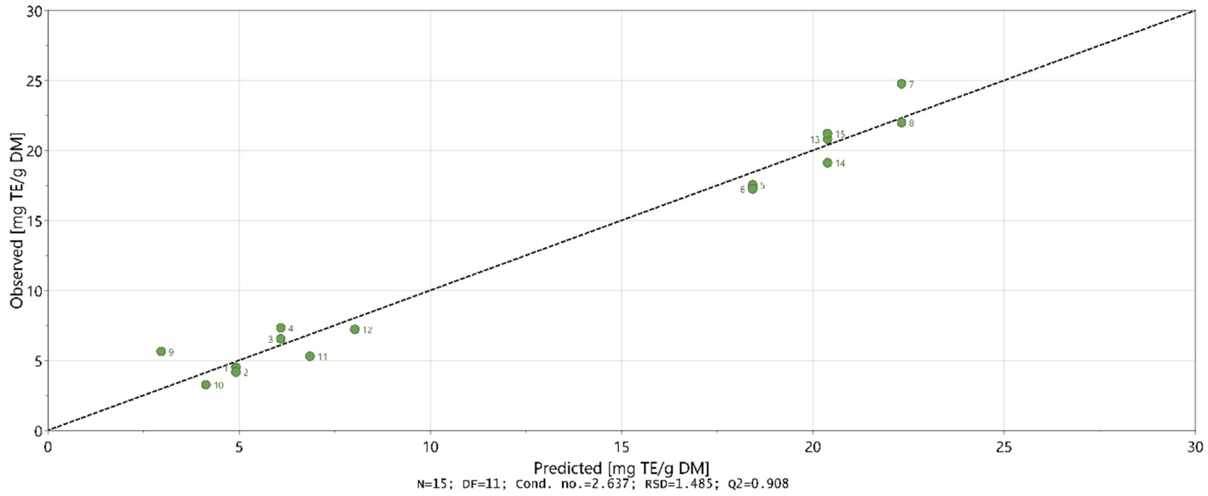

Figure S3. The correlation between the observed and the predicted responses

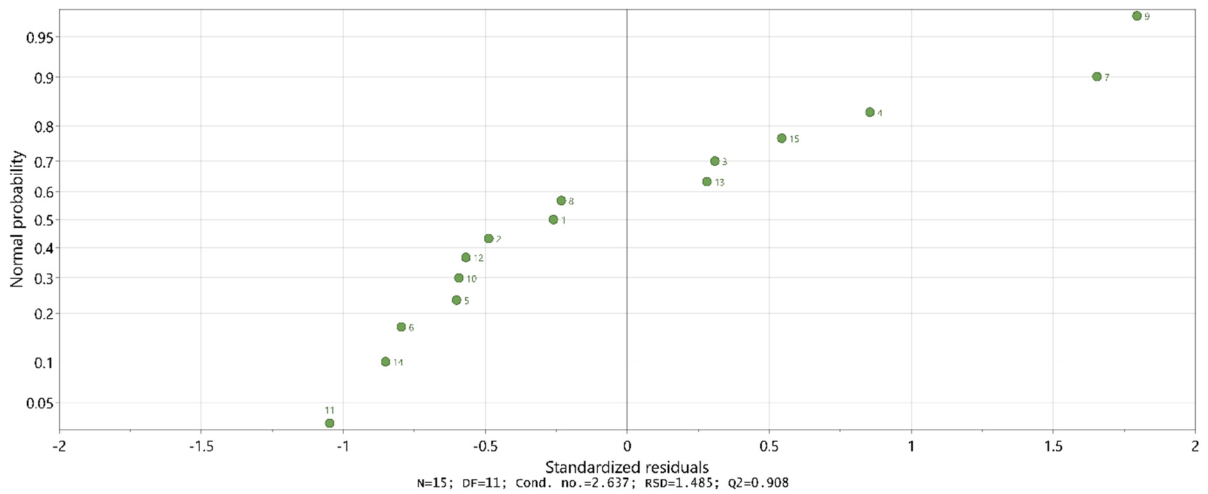

Figure S4. Residual Normal Probability
